# From Normal Cognition to Dementia: Using Natural Language Processing to Identify Cognitive Stages from Clinical Notes

**DOI:** 10.1002/alz.089228

**Published:** 2025-01-03

**Authors:** Samad Amini, You Cheng, Colin G. Magdamo, Ioannis Paschalidis, Sudeshna Das

**Affiliations:** ^1^ Division of Systems Engineering, Boston University, Boston, MA USA; ^2^ Massachusetts General Hospital, Harvard Medical School, Boston, MA USA; ^3^ MassGeneral Institute for Neurodegenerative Disease, Charlestown, MA USA; ^4^ Massachusetts General Hospital, Boston, MA USA; ^5^ MIND Data Science Lab, Cambridge, MA USA; ^6^ Faculty of Computing & Data Sciences, Boston University, Boston, MA USA; ^7^ Department of Electrical & Computer Engineering, Boston University, Boston, MA USA; ^8^ Department of Biomedical Engineering, Boston University, Boston, MA USA; ^9^ Harvard Medical School, Boston, MA USA; ^10^ Massachusetts Alzheimer’s Disease Research Center, Charlestown, MA USA

## Abstract

**Background:**

This study responds to the urgent need for automated and reliable methods to detect cognitive impairments on a large scale. It leverages natural language processing (NLP) techniques to predict dementia and mild cognitive impairment (MCI) using clinical notes from electronic health records (EHR).

**Method:**

Our study used an EHR dataset from Massachusetts General Brigham, which included clinical notes from a 2‐year period (2017‐2018) covering 12 types of patient encounters. Sentence segmentation and keyword‐specific extraction were performed using the NLTK tool, focusing on dementia and activities of daily living, thus providing a comprehensive base for our analysis. Our analysis, designed for classifying cognitive stages into normal cognition, MCI, and dementia, involved three binary classification tasks. We employed two innovative NLP methods based on a transformer‐based language model, the Universal Sentence Encoder (USE). The first, Random Sampling, involved extracting and randomly batching sentences containing relevant keywords, each batch processed through the Universal Sentence Encoder (USE) to generate unique 512‐dimensional embeddings. The second method, Encounter‐based Sampling, grouped sentences by their corresponding note encounter types and keyword categories, creating 24 distinct embeddings for each patient. These methods, leveraging the USE’s deep learning capabilities, provided nuanced approach to classifying cognitive stages, enhancing the predictive accuracy of our model.

**Result:**

We evaluated the performance of various classification tasks on the data containing 531 Normal, 153 MCI, and 229 Dementia subjects. These classifications include classifying between normal cognition and dementia (AUC 97.8% in random sampling, AUC 93.6% in encounter‐based sampling), normal cognition and MCI (AUC 81% in random sampling, AUC 74.6% in encounter‐based sampling), as well as normal cognition and cognitive impairment (i.e., merged MCI and dementia stages; AUC 92.4% in random sampling, AUC 85.4% in encounter‐based sampling). Our NLP‐based approaches also significantly outperforms the baseline model based on the number of relevant sentences by a margin of 6‐13% in AUC in these three classification tasks.

**Conclusion:**

Our study harnessed NLP techniques for enhanced dementia staging accuracy using EHR clinical notes. We introduced a highly accurate, fully automated approach with scalability potential, promising to transform hospital practices in managing clinical notes.

